# Mesenchymal Stem Cells and Metabolic Syndrome: Current Understanding and Potential Clinical Implications

**DOI:** 10.1155/2016/2892840

**Published:** 2016-05-29

**Authors:** Kenichi Matsushita

**Affiliations:** Division of Cardiology, Second Department of Internal Medicine, Kyorin University School of Medicine, Tokyo 181-8611, Japan

## Abstract

Metabolic syndrome is an obesity-based, complicated clinical condition that has become a global epidemic problem with a high associated risk for cardiovascular disease and mortality. Dyslipidemia, hypertension, and diabetes or glucose dysmetabolism are the major factors constituting metabolic syndrome, and these factors are interrelated and share underlying pathophysiological mechanisms. Severe obesity predisposes individuals to metabolic syndrome, and recent data suggest that mesenchymal stem cells (MSCs) contribute significantly to adipocyte generation by increasing the number of adipocytes. Accordingly, an increasing number of studies have examined the potential roles of MSCs in managing obesity and metabolic syndrome. However, despite the growing bank of experimental and clinical data, the efficacy and the safety of MSCs in the clinical setting are still to be optimized. It is thus hoped that ongoing and future studies can elucidate the roles of MSCs in metabolic syndrome and lead to MSC-based therapeutic options for affected patients. This review discusses current understanding of the relationship between MSCs and metabolic syndrome and its potential implications for patient management.

## 1. Introduction

Metabolic syndrome is regarded as a complex cluster of obesity-related complications, and, in recent years, this syndrome has become a global health problem [1–3]. Dyslipidemia, hypertension, and diabetes or glucose dysmetabolism are the major factors constituting metabolic syndrome, and these factors are interrelated and share underlying pathophysiological mechanisms [1–3]. Severe obesity predisposes individuals to metabolic syndrome and affected patients have an increased risk of cardiovascular disease and mortality [1–3].

Recent evidence suggests that mesenchymal stem cells (MSCs) are a major source of adipocyte generation, resulting in an increased adipocyte number. MSCs can be derived from a variety of adult tissues such as bone marrow [4], adipose tissue [5], umbilical cord [6], endometrium [7], skeletal muscle [8], pancreas [9], and liver cells [10]. The biology of MSCs and their capacity to treat various diseases have therefore been extensively studied, as well as the potential roles of MSCs in managing the various components of metabolic syndrome [11–15]. Despite the precise nature and functions of MSCs remaining unclear, MSC-based clinical trials are also completed or ongoing. However, therapeutic applications of MSCs in the clinical setting depend on their safety and efficacy, both of which have yet to be optimized. This review discusses current understanding of the relationship between MSCs and metabolic syndrome and its potential implications for the treatment of affected patients using MSCs.

## 2. Adipogenesis from MSCs

Mesenchymal stem cells are multipotent cells with the potential to differentiate into a variety of cell lineages including fat, bone, cartilage, muscle, and marrow stroma [4]. Although adipogenesis is a multistep process involving many cellular intermediates, for practical purposes it is currently characterized in two major phases: the determination phase and the terminal differentiation phase [16–18]. The determination phase involves commitment of a pluripotent stem cell into the adipocyte lineage [16–18]. In the terminal differentiation phase, the fibroblastic preadipocyte takes on characteristics of the spherical mature adipocyte, in that it can synthesize and transport lipids and secrete adipocyte-specific proteins, and it contains the machinery necessary for insulin sensitivity [16–18].

**Figure 1:**
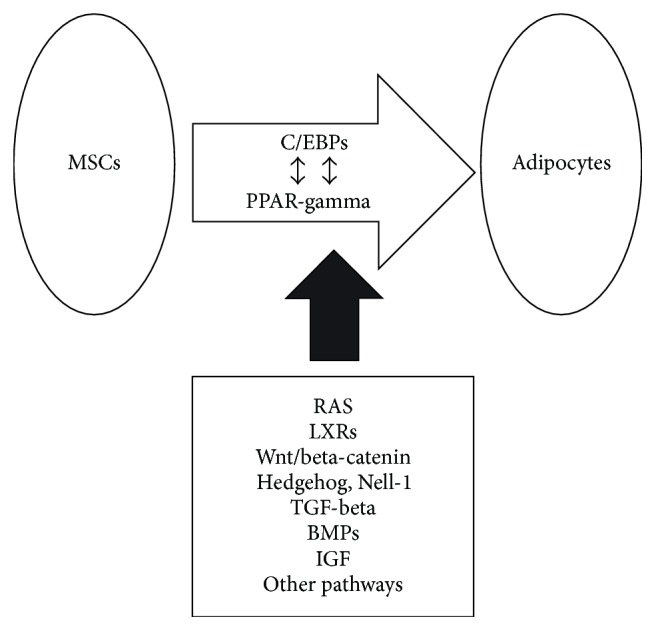
Overview of the established and potential regulatory mechanisms/pathways underlying MSC adipogenesis. A number of cellular signaling mechanisms/pathways control MSC adipogenesis, with the majority converging to regulate a range of transcription factors such as members of the CCAAT/enhancer-binding family of proteins (C/EBPs) and peroxisome proliferator-activated receptor-gamma (PPAR-gamma). Several of these mechanisms/pathways are likely to operate simultaneously; however, the full scope of mechanisms/pathways influencing MSC adipogenesis remains unknown. BMPs, bone morphogenetic proteins; IGF, insulin-like growth factor; LXRs, liver X receptors; MSCs, mesenchymal stem cells; RAS, renin-angiotensin system; and TGF, transforming growth factor.

The signaling pathways governing MSC adipogenesis are numerous and quite complex, with the majority converging to regulate a range of transcription factors such as peroxisome proliferator-activated receptor-gamma (PPAR-gamma) and several members of the CCAAT/enhancer-binding family of proteins (C/EBPs) (Figure 1) [16–20]. Wnt/beta-catenin signaling is one of the most important and well-studied cellular signaling pathways [21, 22] and is also reported to play a pivotal role in the adipogenic differentiation of preadipocytes [23, 24]. Adipogenesis is reportedly blocked by activation of Wnt/beta-catenin signaling but promoted by the inhibition of endogenous Wnt signaling [24, 25], suggesting that Wnts act as a brake for adipogenic differentiation. Specifically, Ross et al. [24] implicated Wnt10b as the most important endogenous regulator of adipogenesis, while Longo et al. [26] showed that transgenic mice in which Wnt10b is expressed from the FABP4 promotor (FABP4-Wnt10b mice) had reduced adiposity and that FABP4-Wnt10b mice were resistant to diet-induced obesity. In addition, Wright et al. [27] transferred a FABP4-Wnt10b transgene onto the* ob/ob* obesity background and demonstrated that expression of Wnt10b in adipose tissue reduces adiposity in the* ob/ob* mouse obesity model. Those authors also crossed FABP4-Wnt10b and lethal yellow agouti (A^y^) mice and showed that Wnt10b protected against genetic obesity in mice due to the ectopic expression of agouti (A^y^) [27].

Other signaling pathways also have important roles in MSC adipogenesis (Figure 1). For example, Hedgehog signaling and Nell-1 signaling are reported to have antiadipogenic effects on MSCs [28–30], while transforming growth factor-beta (TGF-beta) signaling might inhibit adipogenesis from MSCs [31, 32]. Conversely, some bone morphogenetic protein (BMP) family members as well as insulin-like growth factor signaling are reported to promote adipogenesis [33–38]. However, the full scope of signaling pathways and protein interactions influencing MSC adipogenesis remains unknown, and the potential of effective and safe MSC-based therapeutic strategies for obesity relies on ongoing and future studies to further dissect these signaling interactions.

## 3. Obesity and MSCs

Obesity is considered the main culprit of metabolic syndrome. Severe obesity is ascribed to an increase in adipocyte size combined with increased adipocyte number [39–41]. New adipocytes arise from a preexisting pool of adipose stem cells regardless of age [42, 43], and recent studies likened adipose stem cells to bone marrow-derived MSCs [5, 43, 44]. In addition, Liechty et al. [45] demonstrated that human MSCs transplanted into fetal sheep marrow successfully engrafted and differentiated into adipocytes in adult adipose tissue. Moreover, Crossno Jr. et al. [46] showed that adipocyte progenitor cells originating from bone marrow contribute to the development of new adipocytes in adipose tissue. Developmentally, MSCs lie between undifferentiated multipotent embryonic stem cells and adipose tissue-derived preadipocytes, and MSCs are a major cell source for adipogenesis [38, 47, 48]. MSCs are now suggested to play an important role in maintaining the mass and function of adult adipose tissue.

It is well known that exercise suppresses obesity. Interestingly, Rubin et al. [49] hypothesized that low-magnitude mechanical signals would suppress adiposity, not by metabolizing existing adipose tissue but instead by suppressing the differentiation of MSCs into adipocytes. Their experiments showed that 15 weeks of brief, daily exposure to high-frequency mechanical signals, induced at a magnitude well below that encountered during walking, inhibited adipogenesis in mice, and they further demonstrated that irradiated mice receiving bone marrow transplants from heterozygous green fluorescent protein (GFP)^+^ mice showed a reduced commitment of MSC differentiation into adipocytes [49]. The authors thus concluded that formation of adipose tissue in their models was deterred by a marked reduction in MSC adipogenesis and in turn suggested that obesity in humans could be prevented by controlling MSC adipogenesis [49].

Unfortunately, the underlying pathophysiology of obesity remains unclear, and, thus far, the most effective treatment for severe obesity is invasive, bariatric surgery [50–53]. Such surgery to treat obesity might also resolve hypertension, diabetes, and dyslipidemia, resulting in an overall reduction in cardiovascular risk [50–53]. To investigate the mechanism of this surgical treatment of obesity, Chen et al. [54] examined the expression of renin-angiotensin system- (RAS-) related genes in human adipose-derived MSCs and differentiated adipocytes, finding that obese individuals showed upregulation of the RAS-related gene in MSCs and differentiated adipocytes and that such upregulation resolved in post-bariatric surgery subjects. These data together suggest that the treatment of obesity could ameliorate metabolic syndrome, possibly via the modulation of MSC adipogenesis.

## 4. Dyslipidemia and MSCs

Shen et al. [14] reported that high-density lipoprotein (HDL) promotes proliferation of adipose-derived MSCs; however, the exact relationship between cholesterol/lipid homeostasis and MSC biology remains largely unknown. In this context, liver X receptor-alpha (LXR-alpha) is of note. LXR-alpha is an oxysterol-regulated nuclear hormone receptor that plays a central role in cholesterol and lipid homeostasis [55–63]. The expression of LXR-alpha is restricted to tissues related to lipid metabolism, such as the liver, adipose tissue, kidney, small intestine, skeletal muscle, and adrenal gland, whereas LXR-beta is expressed ubiquitously. Recent evidence also reveals an obesity-related effect of LXRs, in that the chronic activation of LXR by its agonist blocked the development of high-fat diet-induced obesity in mice [64]. In addition, accumulating evidence suggests a direct role for LXR-alpha in adipose tissue, with increased expression during adipogenesis [65–67] and high levels in adipocytes [68]. Furthermore, many LXR-alpha target genes are also highly expressed in adipocytes [69].

We recently reported the role of LXR-alpha in adipogenesis of MSCs [70]. Adult murine MSCs (mMSCs) were isolated from the bone marrow of wild type (WT) and LXR-null mice. Using WT mMSCs, we further generated cell lines stably overexpressing GFP-LXR-alpha (mMSC/LXR-alpha/GFP) or GFP alone (mMSC/GFP) by retroviral infection. Compared with MSCs isolated from WT mice, MSCs from LXR-null mice showed significantly increased adipogenesis, as determined by lipid droplet accumulation and adipogenesis-related gene expression. On the other hand, mMSC/LXR-alpha/GFP exhibited significantly decreased adipogenesis compared with mMSC/GFP [70]. Since Wnt/beta-catenin signaling is reported to inhibit adipogenesis, we further examined this signaling nexus. The LXR-null group exhibited significantly decreased Wnt expression accompanied by a decrease of cellular beta-catenin (versus WT), whereas the mMSC/LXR-alpha/GFP group showed significantly increased Wnt expression accompanied by an increase in cellular beta-catenin (versus mMSC/GFP) [70]. Our data thus demonstrated that LXR-alpha has an inhibitory effect on adipogenic differentiation in mMSCs with Wnt/beta-catenin signaling, although the clinical relevance of the antiadipogenic effect of LXR-alpha on MSCs remains unknown. Importantly, LXR-null mice bred onto the OB background (*ob/ob* LXR-alpha-beta^−/−^ mice) show increased total body adiposity compared with WT OB mice [71]. On the other hand, LXR-null mice show resistance to diet-induced obesity through increased energy expenditure [72]. So far, adipose tissue-specific LXR-null mice have not yet been generated; thus, it is difficult to identify the precise roles of LXR in adipose tissue in vivo, and further studies are needed to elucidate the involvement of LXR in MSC adipogenesis and adipose biology.

## 5. Hypertension and MSCs

The RAS is one of the most important factors in the development of hypertension. Previously, we reported that human bone marrow-derived MSCs express all RAS components and that endogenous angiotensin II (Ang II) production is increased in human MSCs undergoing adipocyte differentiation via increased local renin expression [73]. We also demonstrated that Ang II inhibits adipocyte differentiation of human MSCs associated with an increase in type 2 receptor [73] and that blocking this receptor promotes adipogenesis and inhibits osteogenesis of human MSCs [73, 74]. Since the RAS is considered central to the pathophysiology of hypertension, a link between MSC adipogenesis and metabolic syndrome is thus proposed, although such a relationship seems to be quite complex. We also reported that mMSCs could develop into renin-secreting granular cells under the activation of LXR-alpha [75]. However, since renin is the first and rate-limiting step of the RAS and LXR-alpha plays a vital role in the pathophysiology of dyslipidemia, the link between RAS and LXR-alpha in MSCs [76] further complicates the role of RAS in the relationship between MSC adipogenesis and metabolic syndrome. In addition, previous reports related to preadipocytes and the RAS are limited and inconsistent, with some showing Ang II promoting adipogenesis of rodent preadipocytes via the AT2 receptor-mediated formation of prostacyclin [77, 78], while another demonstrated that Ang II reduced adipogenesis of human preadipocytes [79]. In addition, Janke et al. [80] reported that Ang II inhibited adipogenesis of human preadipocytes via the AT1 receptor. These inconsistent data may reflect differences in species, cell types, and culture conditions. Indeed, Chen et al. reported that Ang II inhibited adipogenesis of human adipose-derived MSCs [54], consistent with our results [73]. In this context, the possibility of a two-step process for Ang II's regulation of adipogenesis from stem cells to preadipocytes to adipocytes is also suggested, and further studies are needed to better elucidate the roles of the RAS in MSC adipogenesis.

Besides adipogenesis, the relationship between MSC and hypertension has also been explored. Marketou et al. [13] reported that patients with hypertension had increased circulating MSCs compared with normotensive patients, while de Oliveira et al. [81] recently demonstrated that priming of MSCs with endothelial growth medium prior to intraperitoneal injection significantly induced a prolonged reduction in arterial pressure with improved endothelial dysfunction in an experimental model of spontaneously hypertensive rats. The latter authors suggested that MSCs could improve endothelial dysfunction in the hypertensive state [82, 83] and thus represent a promising alternative to conventional therapy for hypertension [81]. Although the use of MSCs as a therapeutic challenge in hypertension is still in its infancy [84], these cells have demonstrated potential as novel drug targets in therapeutic strategies for hypertension.

## 6. Diabetes and MSCs

Obesity is a leading cause of insulin resistance and type 2 diabetes; thus the connection between adipogenesis and type 2 diabetes is evident [12, 85]. Therapies that can control MSC adipogenesis may reduce the onset of type 2 diabetes; however, further studies are needed to understand the precise mechanisms linking these two conditions.

In addition to the control of adipogenesis, MSCs show potential for the treatment of diabetes. To this end, Chandra et al. [86] showed that murine adipose-derived MSCs differentiated into pancreatic hormone-expressing islet-like cell aggregates, while Marappagounder et al. [87] demonstrated that human bone marrow-derived MSCs differentiated into pancreatic islet-like clusters. Several groups also reported that human adipose-derived MSCs could differentiate into insulin-producing cells [88–91], suggesting MSCs as a source of transplantation material in the treatment of diabetes. In addition, Ohmura et al. [92] showed that cotransplantation of an allogenic islet graft with autologous adipose-derived MSCs under the kidney capsule promoted animal survival and insulin function of the graft, while Fumimoto et al. [93] also demonstrated that implantation of adipose-derived MSCs combined with minced adipose tissue enhanced subcutaneous grafting of islets in diabetic mice. Both these latter studies thus demonstrated the angiogenic and anti-inflammatory potential of MSCs in supporting islet transplantation [92, 93]. Intravenous injection of adipose-derived MSCs also decreased fasting blood glucose levels and suppressed pancreatic islet damage in streptozocin-induced diabetic rats [94] and decreased blood glucose levels and increased glucose tolerance in a high-fat diet-induced obese mouse model [95]. Finally, a clinical trial exhibited that coinfusion of in vitro-generated insulin-secreting cells differentiated from autologous adipose-derived MSC and bone marrow-derived hematopoietic stem cells into the portal circulation, thymus, and subcutaneous tissue increased serum C-peptide levels and improved glycosylated hemoglobin levels [96]. Although there are still unresolved concerns about the safety and the efficacy of stem cell therapy, these data suggest that MSCs are a promising therapeutic option for treatment of diabetes in the future.

## 7. Epigenetics

Recently, epigenetics has provided new insights into the mechanisms of MSC adipogenesis and obesity. Epigenetic mechanisms affect the regulation of gene expression without changing the DNA sequence and include DNA methylation, histone modification, and various RNA-mediated processes [97, 98]. In particular, microRNAs, which are endogenous 18- to 25-nucleotide-long noncoding RNAs that regulate gene expression at the posttranscriptional level, have emerged as important epigenetic players [99], and expression levels of the microRNA miR-148a were shown to be increased in adipose tissue from obese people and mice fed a high-fat diet [100]. These authors showed that miR-148a acted by suppressing its target gene, Wnt1, an endogenous inhibitor of adipogenesis, and, indeed, ectopic expression on miR-148a in human adipose-derived MSCs accelerated adipogenesis and partially rescued Wnt1-mediated inhibition of adipogenesis [100]. Furthermore, knockdown of miR-148a in the same cells inhibited adipogenesis [100]. These data thus implicated miR-148a as a potential biomarker of obesity and promoter of MSC adipogenesis through the repression of Wnt1 signaling [100]. Wang et al. [101] also reported the importance of obesity-associated miR-342-3p in MSC adipogenesis, showing enrichment of this microRNA in the adipose tissue of obese mice and significantly increased expression during adipogenic differentiation in both human adipose-derived MSCs and murine 3T3L1 cells. Overexpression of miR-342-3p promoted adipogenesis of human adipose-derived MSCs, whereas inhibition of miR-342-3p blocked adipogenesis of MSCs [101]. The authors further demonstrated that miR-342-3p directly targeted and inhibited the expression of CtBP2 (a corepressor of the initial factor, C/EBP-alpha) at the posttranscriptional level [101]. On the other hand, inhibition of CtBP2 promoted adipogenesis by releasing C/EBP-alpha and activating adipogenic marker genes, suggesting that miR-342-3p might be a potential target for the management of obesity and other metabolic diseases [101].

Although limited data are available about epigenetic mechanisms associated with the relationship between obesity and MSC adipogenesis, Boyle et al. recently reported that MSCs from infants born to obese mothers exhibited greater potential for adipogenesis [102]. In that study, umbilical cord-derived MSCs from infants born to obese mothers exhibited lower beta-catenin protein content and increased adipogenesis compared with MSCs from infants born to normal-weight mothers [102]. In addition, inhibitions of glycogen synthase kinase-3beta by lithium chloride increased the nuclear beta-catenin content and normalized nuclear PPAR-gamma in MSCs from infants born to obese mothers. The authors further demonstrated that oil Red O staining in adipogenic differentiating cells was positively correlated with the infant's percentage of body fat, leading to the conclusion that maternal obesity might have important consequences for adipogenesis in MSCs of infants and for pediatric obesity risk [102].

Currently, the epigenetic mechanisms underlying MSC adipogenesis, obesity, and obesity-related consequences such as metabolic syndrome are only beginning to be understood. Further studies will hopefully further decipher the epigenetic mechanisms at play in MSC adipogenesis and obesity, leading ultimately to novel epigenetic-based therapeutic options for patients with metabolic syndrome.

## 8. Pitfalls

Since obesity represents an excessive accumulation of adipose tissue, genetic interventions or pharmacologic therapies to reduce adipogenesis might seem attractive approaches to obesity and metabolic syndrome. However, simple inhibition of adipogenesis alone is not an appropriate approach to managing these conditions. Obesity derived from an imbalance between energy intake and output must be taken into account, particularly in strategies to modulate adipogenesis [20], and simply inhibiting the requisite adipogenesis would require an alternative site for the excess calories. For example, Moitra et al. [103] used adipocyte-specific expression of a dominant-negative protein transgene to ablate adipose tissue growth and differentiation, generating a transgenic mouse with no white adipose tissue throughout life. The resultant transgenic mice were initially growth delayed but, by week 12, surpassed their littermates in weight, showing a liver engorged with lipid and enlarged internal organs [103]. Furthermore, the mice were diabetic, with reduced leptin and elevated serum glucose, insulin, free fatty acids, and triglycerides, leading the authors to propose their transgenic mouse as a model for human lipoatrophic diabetes [103]. In this context, a possible relationship between MSCs and ectopic lipid accumulation is an important topic. Ectopic lipid accumulation in insulin-responsive tissues such as skeletal muscle and liver is associated with insulin resistance, type 2 diabetes, and adverse metabolic phenotypes [104–108]. Skeletal muscle is regarded as the main destination for insulin-stimulated glucose disposal and individuals with insulin resistance have increased intramuscular lipid content compared to insulin-sensitive control subjects [109–111]. Fat deposition in the liver can induce hepatic insulin resistance and precede the development of type 2 diabetes [104, 106, 108, 112]. In addition, recent evidence suggests that ectopic lipid accumulation in pancreas could contribute to beta-cell dysfunction [105, 113–115]. Heni et al. [113] demonstrated a negative association between pancreatic fat and insulin secretion that might lead to beta-cell dysfunction, while Szczepaniak et al. [114] reported that the higher lipid content in pancreas might be associated with greater beta-cell lipotoxicity and thus a reduced ability to enhance compensatory insulin secretion. One potential role of MSC adipogenesis in fat tissue might be to reduce ectopic lipid accumulation. On the other hand, skeletal muscle, liver, and pancreas also contain MSCs [8–10, 116]. Although there are limited data linking MSCs with ectopic lipid accumulation, Uezumi et al. [117] showed that mesenchymal progenitors located in the muscle interstitium were the major contributor to ectopic fat cell formation in skeletal muscle. The relationship between MSCs and ectopic lipid accumulation is an area deserving of more research and further studies are warranted.

In general, failure to produce new adipocytes leads to an increase in large insulin-resistant adipocytes and a predisposition to developing diabetes and the metabolic syndrome [118]. Therefore, promoting new, small, and insulin-sensitive adipocytes would be expected to yield medical benefits. On the other hand, Rieusset et al. [119] reported that a PPAR-gamma-specific antagonist protected mice from high-fat diet-induced adipocyte hypertrophy and insulin resistance by preventing adipocyte differentiation and lipid accumulation. In adipose tissue, the possibility exists that decreased PPAR-gamma expression improved insulin sensitivity, as demonstrated in PPAR-gamma-deficient mice [120, 121] or treatment with a PPAR-gamma-specific antagonist [119]. However, it must be acknowledged that conflicting data exist as to the effects of PPAR-gamma activity, its agonist, and its antagonist on obesity and diabetes [119–124]. Indeed, thiazolidinediones, which are synthetic activators of PPAR-gamma, are used clinically to reduce hyperglycemia in the treatment of patients with type 2 diabetes [125]. An appropriate balance between promoting requisite adipogenesis and inhibiting excess adipogenesis might therefore be important in maintaining a caloric equilibrium.

In this regard, brown and white adipocytes are of note. Mammals contain two types of adipose tissue: white adipose tissue (WAT) and brown adipose tissue (BAT) [126]. WAT is involved in energy storage, whereas BAT has a unique thermogenic capacity resulting from the expression of uncoupling protein 1 in the mitochondrial inner membrane [126, 127]. Importantly, BAT activation-regeneration in animal models reduces obesity and improves insulin sensitivity, and the fat-burning activity of BAT might be exploited to develop a novel therapeutic option for the treatment of obesity and metabolic syndrome [127–131]. Furthermore, recent studies suggest that another type of brown adipocytes, called beige or bright adipocytes, with similar functions to those of brown adipocytes could exist in WAT and that prostaglandin E2 signals could induce white-to-brown adipogenic differentiation [126, 132, 133]. However, the mechanisms underlying BAT activation-regeneration remain ill defined, and, in addition, genetic or pharmacologic interventions to manipulate BAT activation-regeneration are currently not well controlled, with the possible adverse effects still unclear [127]. Further studies are needed to ascertain whether BAT activation-regeneration could be efficacious in humans and thus possibly underlie a novel therapeutic strategy for obesity and metabolic syndrome.

## 9. Future Perspectives

**Figure 2:**
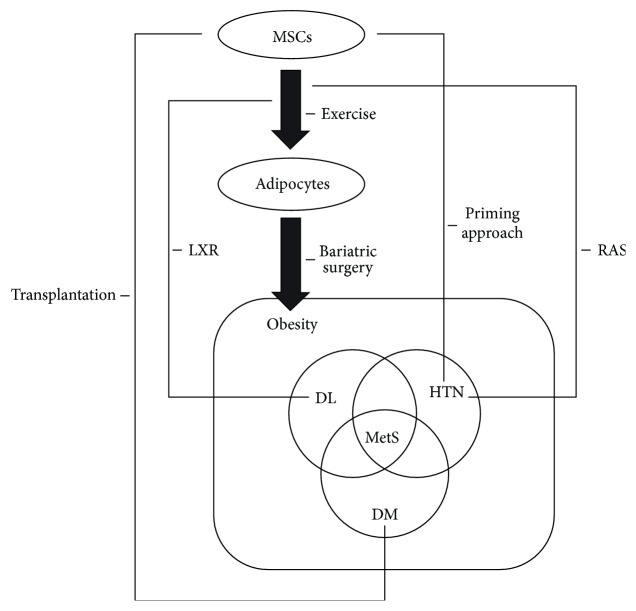
Schema of the clinical impact of MSC adipogenesis on metabolic syndrome. Pathophysiology of metabolic syndrome is quite complex and the precise mechanisms linking each condition remain unclear. Among the potential clinical applications of MSCs, studies assessing MSCs as a source of transplantation material in the treatment of diabetes have shown encouraging results. MSC-based therapeutic options for diabetes could be used in the clinical setting in the future. Although further studies are needed to elucidate the roles of MSCs and MSC adipogenesis in metabolic syndrome, MSC therapy is expected to become a new level of therapeutic option for this syndrome. DL, dyslipidemia; DM, diabetes mellitus; HTN, hypertension; LXRs, liver X receptors; MetS, metabolic syndrome; MSCs, mesenchymal stem cells; and RAS, renin-angiotensin system.

An improved understanding of how MSC adipogenesis relates to each condition constituting metabolic syndrome is clearly of utmost importance for possible clinical translation. Adipogenesis itself is a highly complex process. In addition, the relationship between adipogenesis and obesity is associated not only with cellular differentiation but also with the modulation of inflammatory cytokines and cellular processes such as mitochondrial biogenesis. Furthermore, the pathophysiology of metabolic syndrome is complex and the precise mechanisms linking each condition remain unclear, limiting the current therapeutic options; thus the potential roles of MSCs in the treatment of metabolic syndrome hold significant promise for patients (Figure 2). Further clinical studies are now needed to resolve the safety and efficacy issues surrounding the clinical applications of MSCs, with larger populations of patients and longer monitoring durations. In the future, MSC therapy is expected to become a new level of therapeutic option for metabolic syndrome.

## 10. Conclusions

Metabolic syndrome is an obesity-based, complicated clinical condition that has become a global epidemic problem. The underlying pathophysiology of metabolic syndrome remains ill defined and current therapeutic options for patients with this syndrome are limited. Since MSCs are suggested to be a major source of adipocyte generation, studies are ongoing into the potential roles of MSCs in the management of obesity and metabolic syndrome and into their safety and efficacy in the clinical setting. Further elucidation of MSC biology and the roles of MSCs in metabolic syndrome could lead to the development of effective MSC therapy for patients with this syndrome.
